# Promoting men-inclusive maternity services: exploring the expectations, experiences and needs of men as fathers

**DOI:** 10.1186/s12884-024-06644-3

**Published:** 2024-07-12

**Authors:** Gai Harrison, Kristy Fitzgerald, Patrick O’Leary, Alka Kothari, Leonie Callaway

**Affiliations:** 1 The Royal Brisbane and Women’s Hospital, Herston, QLD Australia; 2 School of Health Sciences and Social Work, Griffith University, Logan, QLD Australia; 3 Redcliffe Hospital, Anzac Avenue, Redcliffe, QLD Australia; 4 Faculty of Medicine, The University of Queensland, St Lucia, QLD Australia; 5 School of Nursing, Midwifery and Social Work, The University of Queensland, St Lucia, QLD Australia

**Keywords:** Men, Fathers, Maternity services, Inclusion

## Abstract

**Aim:**

This study aimed to explore the ‘real time’ expectations, experiences and needs of men who attend maternity services to inform the development of strategies to enhance men’s inclusion.

**Methods:**

A qualitative descriptive design was adopted for the study. Semi-structured face-to-face or telephone interviews were conducted with 48 men attending the Royal Brisbane and Women’s Hospital before and after their partner gave birth. Data were coded and analysed thematically.

**Results:**

Most respondents identified their role as a support person rather than a direct beneficiary of maternity services. They expressed the view that if their partner and baby’s needs were met, their needs were met. Factors that contributed to a positive experience included the responsiveness of staff and meeting information needs. Factors promoting feelings of inclusion were being directly addressed by staff, having the opportunity to ask questions, and performing practical tasks associated with the birth.

**Conclusion:**

Adopting an inclusive communication style promotes men’s feelings of inclusion in maternity services. However, the participants’ tendency to conflate their needs with those of their partner suggests the ongoing salience of traditional gender role beliefs, which view childbirth primarily as the domain of women.

**Supplementary Information:**

The online version contains supplementary material available at 10.1186/s12884-024-06644-3.

## Background

There is growing recognition of the importance of involving men in antenatal care and childbirth to optimise outcomes for maternal and newborn health [1, 2]. While fathers are increasingly expected to be actively involved with antenatal care and present at the birth [3], they commonly report feeling excluded or marginalised during antenatal appointments and childbirth [4–6]. Programs promoting father-inclusion tend to focus on how men can best support their partners rather than addressing their expectations and needs [7, 8]. Viewing men predominantly as support persons or bystanders during pregnancy and childbirth precludes a more holistic understanding of their needs [9, 10]. In consequence, the culture of maternity services has not evolved to incorporate men’s presence in a way that signifies meaningful inclusion [11, 12].

Expectant fathers commonly report uncertainty and anxiety [13], while first-time fathers report feeling unprepared for the emotional, relational and practical challenges associated with becoming a father [14, 15]. A pre-existing mental health diagnosis, low household income and low levels of social support can intensify pregnancy-related challenges [16]. Men’s developmental histories, such as early adverse events and trauma, can be a trigger and impact how they perceive their capability and needs in becoming a father. Yet, there is little or no opportunity to seek assistance at procedures such as ultrasounds where such triggers may occur [17]. There is also a lack of evidence regarding how services can better engage expectant fathers experiencing stress and anxiety [18]. Where these men are identified, it tends to be due to the perceived risk they pose to the mother and baby, which may result in them being excluded from care planning, leading to all responsibility being left with the mother [19].

Men who subscribe to traditional views of masculinity may deny their needs during childbirth, aided by the belief that attention should be focused on their partners who are giving birth [6, 11, 20]. Of further concern is the finding that fathers may believe that they are not entitled to health professionals’ support [11, 21]. While several studies indicate that men’s need for information takes priority over other needs associated with the birth experience [8, 9, 22], those who adhere to traditional constructs of masculinity may minimise their need for emotional support [7]. Other researchers have highlighted the importance of exploring how different sociocultural frames of reference shape men’s understanding of fatherhood and their role in the birth [7, 23–25].

The literature highlights the gendered nature of becoming a parent within patriarchal structures. These structures have the dual effects of exempting men from taking equal responsibility for caring for a baby while at the same time isolating fathers from being involved in the baby’s care. Hegemonic masculinity as conceptualised by Connell (1995) has been seen as a process that marginalises men from being active participants in healthcare services in the context of pregnancy [26]. This operates both in the way health professionals perceive men’s needs and behaviour, and how men engage with their partner and health services [27]. Research has highlighted that fathers perceive health professionals as lacking skills to effectively engage them [28] and that hospitals lack facilities to cater for their needs [29]. At a practical level, work schedules and childcare responsibilities may also prevent men from attending antenatal appointments [30, 31]. Researchers have therefore called for a focus on fathers’ needs and the barriers they face since current healthcare systems do not respond effectively to men’s needs as they face parenthood [32].

More recently, attention has focused on how initiatives to increase men’s engagement in maternity services may inadvertently disempower some women, including those in abusive relationships [33]. Domestic violence during pregnancy is well documented, although the effectiveness of maternity services in identifying and engaging with expectant fathers posing a risk of violence lacks a strong evidence base [34]. Given these concerns, it has been suggested that interventions to increase men’s participation in maternity care need to be ‘gender transformative’ to guard against reinforcing existing power imbalances [33]. Although minimal guidance is available on how this transformation should take place, advocates of father-inclusive practice promote a model of service delivery underpinned by the principles of recognising and responding to the diverse needs and circumstances of men while balancing their needs with those of the family as a whole [30, 35]. As a first step in this process, organisations are encouraged to undertake an audit to determine how well their programs deliver father-inclusive services, focusing on access, equity, communication, and responsiveness [35].

In 2019, an environmental audit of Maternity Services at the Royal Brisbane and Women’s Hospital identified locations where men are likely to accompany their partners, namely: outpatient departments, antenatal classes, maternity wards, and the nursery. The research team also documented and photographed posters, pamphlets, artwork and signage on display in these locations. The audit revealed that men are not often visually represented at most of these sites and when they are it is predominantly as either supports for their partners or perpetrators of violence (e.g., domestic violence posters). Visual materials focusing on fathers’ needs and experiences as men were scarce. Signage similarly omitted reference to men.

Building on these findings, the current study aimed to explore men’s perceptions of their needs and whether they experience a sense of inclusion while attending maternity services. The results would inform a plan for instituting cultural change to enhance men’s sense of inclusion in maternity services.

## Methods

### Design

The aim of this study was to explore men’s subjective expectations and experiences of maternity services in ‘real time’, which refers to the time a person has attended a health service when the experience is still fresh in their mind [36]. A qualitative descriptive design was chosen as it allows for the collection of data in a natural setting and privileges subjective interpretations of events [37]. Semi-structured interviews were used as they are advantageous for exploring a predetermined set of questions while allowing respondents to expand on additional topics they identify as important [38]. Interview guides with prompts were developed to explore men’s perceptions of the environment of Maternity Services and whether they experienced a sense of inclusion, as well as their expectations and experiences of antenatal care and childbirth (see Additional file 1). The use of ‘grand tour’ questions [39], such as *Tell me about your experience of the birth* enabled participants to guide the direction of the interview and expand on their own understandings of inclusion as well as aspects of maternity care that were important to them. When exploring the men’s perceptions of inclusion, prompts focusing on the physical environment, visual representations of men, interactions with staff, information provision, antenatal classes and parent education were used.

Two final year social work students on clinical placement in Maternity Services piloted the interview guide under the supervision of the research team. Both students had completed training on qualitative data collection and conducting interviews. The pilot interviews were observed by a member of the research team to ensure that the students were using appropriate interviewing skills, including establishing rapport, posing open-ended questions, and follow up prompts. Based on feedback from the pilots, an additional prompt was added that focused on whether the men felt they were welcomed in Maternity Services.

### Setting, sample and recruitment

The study was conducted in Maternity Services at the Royal Brisbane and Women’s Hospital, a large tertiary public hospital in Brisbane, Australia, where approximately 4,500 babies are born each year. The partners of women attending maternity services who self-identified as expectant fathers and were ≥ 18 years of age were invited to participate in the study, with representation sought from both first-time fathers and those attending for subsequent births. Exclusion criteria applied to men who had entered a surrogacy arrangement, experienced stillbirth or whose babies had a poor prognosis, or whose partners experienced post-birth complications.

Data collection took place from July to September 2020. Participants were recruited from the maternity outpatients’ department, antenatal classes, screening appointments, the maternity wards, and the nursery. Recruitment was largely opportunistic due to men’s variable attendance patterns at maternity services. Two social work students on clinical placements attended these venues every day for eight weeks to distribute information about the study and oversee the consent process. Men who consented to participate in the study were then interviewed by the students in a private consult room or an alternative private space nominated by the participant. Interview times varied and lasted from 20 to 45 min. Permission was sought from participants to audiotape the interview. Three participants declined to be recorded, including one who was concerned that “his English wasn’t very good”. Another participant asked the interviewer to turn off the digital recorder towards the end of his interview when asked if he had any other comments. In these situations, the interviewer took detailed notes of the participant’s responses, including key direct quotes recorded in italics.

### Ethical considerations

Participants were informed that participation was voluntary, they could withdraw from the study at any point, and that their decision would have no influence on the services they received while at the hospital. The participant information sheet included information on external support services available to the men if they needed to talk to someone after the interview. All data, including transcripts and field notes, were deidentified and each participant was allocated a number. Participants were offered a summary of the findings if they provided a follow up address.

### Analysis

Interviews were transcribed verbatim by a professional transcriptionist and the transcripts were checked for accuracy by a member of the research team. The transcripts were coded and analysed thematically. Two levels of coding were carried out that encompassed both descriptive and pattern coding [40]. First, the data were grouped according to the topics covered in the interview guide. Next, two members of the research team and a social work student working on the project carried out a line-by-line analysis. Drawing on the direct speech of participants, a provisional coding scheme was developed, complemented by the use of analytical memos to pose questions and reflections on the data, including similarities and differences in participants’ responses [41]. The coding scheme was then reviewed by all members of the research team, with the final stage of analysis involving the identification of overarching and sub-themes.

## Results

Forty-eight men participated in the study. Twelve participants were recruited prior to the birth and thirty-six post-birth. They ranged in age from 19 to 48 years. Over half (28) were first-time fathers. Most participants resided in the local metropolitan area, with two commuting from regional cities. The majority (44) were in paid employment. Two participants were unemployed and two were in full time study.

Although data was not collected specifically on gestational age, at the time of interview most partners of the pre-birth cohort were in their third trimester of pregnancy. Potentially, this could have been due to antenatal classes being relocated to the community because of COVID-19 restrictions in place at the time, resulting in fewer presentations to the hospital until later in the pregnancy. Participants in the post-birth cohort were interviewed within one to eight days of the birth.

The analysis identified four key themes. The first theme, *I’m just the support person*, examines the men’s propensity to minimise their role in antenatal care and the birth. The second theme, *If my partner’s and baby’s needs are met, my needs are met*, builds on the first theme to explore the participants’ tendency to conflate their own needs with those of their partner and baby. The third theme, *It was all about the delivery of the information*, highlights the value men placed on meeting their informational needs. The final theme, ‘*How’s Mum, how’s baby, how are you?’* demonstrates how staff who were approachable and used inclusive styles of communication promoted men’s feelings of inclusion in maternity services. These themes are elaborated on below with reference to direct quotes. Each participant (P) is identified by a number.

### I’m just the support person

Overwhelmingly, the participants in this study viewed their role purely as a support person for their partner while attending antenatal appointments and at the birth. This was the case for both first-time fathers and those attending for subsequent births. For example:

P1 *I think, as a male, you’re a passenger, so you’re just there to support. I don’t think I could have been included more.*

Using the analogy of being ‘a passenger’ suggests that inclusion is a secondary concern for this father, who identified his partner as his primary focus. Another participant equated his support role with ‘being strong’ for his partner while also admitting to feeling fearful of caring for a newborn baby. The desire to appear strong stemmed from his need to reduce his partner’s stress and feel useful.

P14 *As a dad, one of the most important things we can do – because there’s not a lot we can do during this time – is take any stress away from the mother…I wanted to be as strong as I possibly could for [partner’s name], but I was also just absolutely terrified of what was on the other side of that blanket.*

Participants identified external factors that hindered their ability to be a supportive presence for their partner, including childcare responsibilities, inflexible work arrangements, and being time poor. Five participants (P2, P3, P4, P5, P15) commented on the amount of time they spent waiting for antenatal appointments, while a lack of timely communication about cancelled appointments was raised as a further concern.

P6 *So the idea was we’d try to [attend appointments] on days that I wasn’t working, so I could go. But if it gets changed last minute, I can’t just leave work like that.*

However, those men who had the option of working remotely or had supportive employers, such as P7 who commented that ‘*work has been accommodating’*, could attend appointments as needed. Accordingly, the men’s ability to support their partners during the antenatal period partly depended on their employment status.

At the time of the birth, many fathers viewed their support role as extending to performing practical tasks associated with their baby’s delivery. Being able to participate in this way appeared to foster feelings of inclusion for the following participant.

P16 *I was involved, because I was the first person the baby went to…because I had to do the weighing and the checking and the cutting of the cord.*

In contrast, another participant voiced his frustration at his inability to be of practical use during the birth, using the analogy of a tradesperson to explain his desire to do concrete tasks.

P17 *You’re not competent, you’re not equipped to do what the midwives are doing, you’re clueless. So that’s why I felt excluded, where as a tradie, you’d love to participate in the work, however this is out of your depth. That’s why I felt excluded in regards to the delivery.*

Along with performing practical tasks, participants saw advocating for their partner as something tangible they could do if they felt that their partner’s needs were not being met. As one father stated:

P18 *I feel this is the Dad’s responsibility to be inquisitive and ask questions and advocate for their partner*.

However, confining their role to support person and advocate meant that the fathers tended to be disengaged from their own needs and primarily focused on the welfare of their partner and child. This theme is explored below.

### If my partner’s and baby’s needs are met, my needs are met

When asked about their expectations of maternity services, most participants either denied having any or conveyed expectations for how their partner or baby should be treated. For example:

P19 *I didn’t have any expectations. I was just making sure that my partner and my child were OK. That was all I had.*

Similarly, when questioned directly about their needs, most participants tended to equate the fulfilment of their needs with the wellbeing of their partner and baby, as exemplified by the following responses.

P20 *I don’t have any needs except for my child being healthy and I know he is in the best place he can be.*

P21 *I’m just along for the ride, as long as they look after Mum and bub, I’m happy.*

Focusing solely on their partner’s wellbeing meant that most of the men struggled to identify their own needs. Despite repeated prompting, one father admitted to drawing a blank when trying to conceptualise his needs. Instead, he reverted to the mantra that his wife was the priority.

P7 *I don’t know what my needs are right now. My wife is most important.*

Alternatively, some participants indicated that they did not expect health professionals to attend to their needs, which suggests they subscribed to the belief that maternity services should focus solely on providing women-centred care.

P2 *I don’t expect them [staff] to take care of my needs because my wife is the one they need to look after.*

Being strongly invested in their partner’s needs being met meant that if men judged their partner’s care to be inadequate, this translated into an unsatisfactory experience of maternity care. This is encapsulated by the following participant’s negative account of his experience of the birth, which he related to his partner’s unmet need for pain relief.

P22 *My experience has been up and down, mostly due to the care of my partner…Doctors did not listen or consider my partner was in pain.*

The men’s tendency to equate their satisfaction with maternity services with their evaluation of how well their partner and child were looked after has been identified in previous research [5]. Yet, despite questioning the legitimacy of their own needs and prioritising their partner’s birth experience, participants articulated a strong desire for information about childbirth. This quest for knowledge is examined in the following section.

### It was all about the delivery of the information

Most participants stressed the importance of being provided with accessible information about antenatal care and the birth. For example, when asked whether anything could have been done differently to improve his experience of Maternity Services, P23 stated: *It was great…For me, it was all about the delivery of the information.* Notably, the provision of information was the defining feature of this father’s positive experience. In particular, being provided with clear explanations about the birth allowed fathers a level of predictability about what was going to happen.

P24 *Everything was very well explained…The doctor was very good, always explaining everything, what they were doing, what was happening, what was going to happen, what likely complications or outcomes of different procedures and things like that.*

In contrast, the use of overly medicalised language limited the men’s understanding of the information they received.

P17 *I wasn’t able to really make sense with all the technical heavy terms….Yeah, the lingo. I think that is a major point I would like to share with you and for the research, dialogue, language for husbands.*

This participant identified the use of language as a ‘major’ factor that shaped his experience, highlighting the importance of maternity clinicians factoring in health literacy when communicating with fathers and tailoring information to their needs.

Some fathers equated the provision of information with inclusion. The following participant related how both he and his partner were given information packs, which signalled to him that his need for information was afforded the same priority as that of his partner.

P7 *I felt included that the staff gave me info as well as my partner, so we understood what was going on.*

The acquisition of information appeared to guard against feelings of helplessness. For example, one participant described how conveying information to his partner provided him with a practical purpose during the birth, which was underpinned by the need to feel like he was doing something useful.

P14 *If I can see all the information and then relay it to her, I feel like that’s really one of the only things I can do.*

Clinicians who could conveyed information in a way that aligned with the men’s needs in the moment were seen in a positive light. In contrast, the following participant described the stress associated with not receiving updates on his partner when she had to undergo surgery. Being placed in a room separate to his partner exacerbated his anxiety.

P25 *I was in a separate room and I wasn’t really getting told much. All I was getting was…’this won’t take long’ and I was, like, ‘Okay I know that, but I still can’t see what’s going on, and I just don’t know what state my wife is in. She’s going in for major surgery’.*

Fathers who were given the opportunity to ask questions about the birth reported favourably on their experience of maternity services, including their interactions with staff.

For example:

P2 *They listened to my questions as well as my partner’s, so I felt included.*

In contrast, another participant described how he felt excluded because when he did ask questions, clinicians directed their responses to his partner.

P8 *Discussions will generally gravitate toward my partner throughout the whole process. I was afforded limited opportunities to ask the questions I wanted but, yeah, the answers were then generally being directed back towards her.*

Being provided with the opportunity to ask questions served the purpose of signalling to the fathers that staff cared about and wanted to hear their concerns. The following participant related how clinicians consistently asked him if he had any questions at antenatal appointments, which made him feel included in consultations.

P9 *They always ask me if I have any questions, every appointment that we went to, which was always good. Even though I don’t have any, it is very good to know they considered what I was thinking as well. So I think, yeah, I felt involved.*

Notably, the simple act of checking in with fathers appeared to serve a symbolic function of demonstrating care on the part of the clinician. The act itself was valued regardless of whether the men had questions to ask, highlighting the importance of interpersonal interactions with maternity staff. This theme is expanded on in the following section.

### How’s Mum, how’s baby, how are you?

Clinicians who were friendly and responsive were seen in a positive light by all participants. Seemingly minor rituals such as engaging in small talk or making jokes were identified as aspects of interpersonal communication that promoted feelings of inclusion.

P10 *… they (staff) come in, you have friendly chats with them all the time. They don’t seem to ignore you or anything. They involve you as much as they can really. I think my experiences here have been pretty awesome.*

Participants frequently commented on how the communication style of clinicians shaped their experiences of maternity services. Being directly addressed by staff appeared to promote feelings of inclusion. This is encapsulated by the following participant’s observation of one midwife who made specific inquiries about his welfare.

P11 *She asked me about four or five times, and that’s even seeing her just walking out the door and she’d stop [and say], ‘how’s Mum, how’s baby, how are you?’ You know, that definitely makes you feel included as a Dad.*

Similarly, P12 and P8 highlighted how staff who took the time to talk with them made them feel included.

P12 T*hey were always really good, talking to me as well as her, not just talking to her. That just made me feel included as well.*

P8 *The high-risk unit we went to was always very good, it was inclusive. To sort of say, you know, ‘How’s Dad going?’, that sort of thing.*

In contrast, P11 observed that ‘S*ome midwives just completely ignored me and…when I’m trying to talk, trying to make conversation they don’t want to hear it’.* As a result, his experience of Maternity Services had been coloured by these negative interactions.

Another father conveyed how he felt consistently supported by the clinicians he encountered, making specific reference to their friendliness and how they were able to contain his stress during the birth.

P26 …*they were the nicest people you could meet and yeah, they kept my nerves calm, so, yeah, the doctors and the nurses there are really good…they supported me, you know, when I wasn’t calm and they kept me calm.*

Interestingly, the clinician’s gender did not appear to be a factor that influenced the men’s assessments of their interpersonal interactions with staff. The following participant reflected on how he could not build a rapport with a male midwife he met on the ward.

P8 *In this obviously heavily female dominated realm, you know I’ve seen one male midwife in my whole time and, even then, I didn’t necessarily feel I could have a chat to him.*

While the quality of clinician-patient communication to patient outcomes is well recognised, less attention has been focused on the calibre of communication between maternity clinicians and fathers. Participants repeatedly commented on the approachability and responsiveness of staff as being key factors that shaped their experiences of maternity services. An indifferent or negative interaction with staff made the men feel excluded, while staff who affirmed their presence and directly addressed them fostered a sense of inclusion.

## Discussion

Unlike previous studies where men reported feeling excluded from maternity care [4–6], the participants in this study provided predominantly positive accounts of their experiences attending antenatal appointments and childbirth. The thematic analysis identified four key themes. Theme one (*I’m just the support person)* demonstrated that participants did not expect health professionals to attend to their needs as expectant fathers, which mirrors previous studies that found men viewed their partners as the legitimate focus of care [11, 21]. Similarly, theme two (*If my partner’s and baby’s needs are met, my needs are met)* highlighted the men’s tendency to equate their satisfaction with maternity services with their evaluation of how well their partner and child were looked after, which has been identified in previous research [5]. This in part explains the men’s positive accounts of attending antenatal appointments and childbirth, as they did not see their needs as a focus for services.

Although constructs of masculinity are subject to ongoing change, these findings suggest that the fathers in this study mostly endorsed traditional gender roles that view men as protectors of the family. As a result, they highlighted the importance of staying strong for their partners and advocating for them if they believed their care was less than optimal. Performing concrete practical tasks also fulfilled the men’s need to feel useful during the birth. Notably, the physical hospital environment was not identified as a factor that hindered feelings of inclusion despite a prior environmental audit revealing the lack of visual materials focusing on men’s needs and experiences.

The fathers in this study placed considerable importance on the provision of information about antenatal care and childbirth. Theme three (*It was all about the delivery of the information*) illustrated how maternity clinicians who provided clear explanations that were pitched at a level commensurate with the father’s level of health literacy were positively evaluated by participants. Access to information allowed for a level of predictability about what was going to happen before, during and after the birth. In contrast, a lack of information fostered feelings of powerlessness and anxiety. While men’s need for information has been highlighted in earlier research [8, 9, 22], the provision of information in this study was also equated with inclusion. Additionally, the opportunity to ask questions was highly valued, as it indicated that clinicians were interested in the men’s concerns.

Overall, the findings of this study underline the importance of the relational dimensions of maternity care, whereby seemingly minor aspects of a consultation with a clinician can shape people’s experiences of a service, exemplified by theme four (*How’s Mum, how’s baby, how are you?).* Encounters with maternity staff that were rated positively by men were equated with inclusion. Building the capacity of clinicians to engage with fathers is a recognised facilitator of father inclusion [30]. The findings from this study suggest that maternity staff who adopt an inclusive style of communication and take time to build rapport with men are more likely to foster their sense of involvement in maternity care. This includes non-verbal gestures such as an attentive look or making eye contact, which similarly signal acknowledgement and an interest in the man’s experience. Inviting men to ask questions is a concrete strategy to promote inclusion as it sends the message that staff are interested in them and, importantly, legitimises their concerns. Offering fathers a separate information pack dedicated to their specific concerns may similarly promote feelings of inclusion.

Relationship-based care has long been recognised as a key factor in improving patient outcomes [42]. Expanding this concept to encompass expectant fathers will potentially enhance their involvement with maternity services and improve maternal and newborn health outcomes. Training clinicians on inclusive styles of communication for engaging fathers is a practical intervention that, from an organisational perspective, is not time or resource intensive. Moreover, ensuring maternity staff are on board is a first step in instigating broader cultural change to embed father inclusion as standard practice.

### Strengths and limitations

A core strength of this study is its depiction of men’s experiences of maternity services in real time, as opposed to retrospective studies that rely on memory recall. The study’s main limitation is the use of a convenience sample, which means the findings cannot be generalised beyond the sample group and the hospital setting where the study was conducted. However, as the purpose of the research was to inform an institution-specific plan for cultural change to enhance men’s inclusion in maternity services, the issue of generalisability to other settings is less relevant.

The exclusion of younger fathers and those men who had experienced a traumatic birth or neonatal death is a further limitation of the study. In addition, although real time data allows access to a person’s immediate recent experience of healthcare, it tends to provide a snapshot rather than reflecting the totality of their experience [43]. The opportunistic recruitment of participants in a busy Maternity Department also meant that some interviews were relatively short (20 min), which could have compromised the richness of the data. Potentially, engaging more experienced researchers rather than students to conduct the interviews could have resulted in the collection of more in-depth data.

The analytical process in qualitative research invariably involves an element of subjectivity. While the research team were mindful of guarding against preconceived ideas about inclusion when analysing the data, the way interview questions are framed and codes are developed entail subjective choices [44]. These choices reflect underlying beliefs and assumptions about key concepts. For example, the research team viewed inclusion as a positive goal to be pursued, which in turn shaped the interpretive process. However, this belief may not necessarily be shared by others, including the participants.

## Conclusion

The men in this study consistently identified interpersonal interactions with clinicians as the most significant factor that shaped their experiences of maternity services. Encouraging clinicians to use inclusive style of communication will assist in building a father-inclusive environment in maternity services. The study further highlighted the men’s adherence to traditional gender role beliefs which view childbirth primarily as the domain of women. Although most reported positive experiences and did not report exclusion, as expectant fathers they did not see their needs as a legitimate focus of care, instead prioritising the needs of the mother and baby. These results point to the need for exploring men’s perceptions of what needs they can raise with healthcare professionals while attending maternity and childbirth services, which in turn can inform future service delivery and evaluation. Future research should also target a larger representative sample that includes men from culturally and linguistically diverse backgrounds and explores how different cultural frames of reference for fatherhood may influence their interactions with maternity services.

### Electronic supplementary material

Below is the link to the electronic supplementary material.

Supplementary Material 1

Supplementary Material 2

## Data Availability

The dataset from this study is not publicly available due to the need to protect the participants’ privacy and confidentiality. Data are available from the corresponding author upon reasonable request and subject to approval from the ethics committee.

## Declarations

### Authors’ information

GH is a clinical educator and qualitative researcher who holds an honorary senior lecturer position in social work. PO is a Professor of Social Work who has extensive experience in both research and clinical work with men and families, focusing on issues such as masculinity, trauma, and men’s use of violence. AK is an Associate Professor in Obstetrics and Gynaecology whose work focuses on policy change and healthcare leadership in the provision of holistic, equitable and compassionate care to fathers and partners experiencing traumatic events during pregnancy and birth. KF is an advanced social worker with extensive clinical experience in maternity and neonatology services. LC is a Professor of Medicine and has supervised and co-authored research regarding men in maternity services in her capacity of Director of Research within Women’s and Newborn services.

## References

[1] World Health Organisation. Fatherhood and health outcomes in Europe. Copenhagen: WHO.2007. https://apps.who.int/iris/bitstream/handle/10665/108571/E91129.pdf. Accessed 3 Jan 2023.

[2] Kothari A, Thayalan K, Dulhunty J, Callaway L (2019). The forgotten father in obstetric medicine. Obstetric Med.

[3] Johansson M, Fenwick J, Premberg Å (2015). A meta-synthesis of fathers׳ experiences of their partner׳s labour and the birth of their baby. Midwifery.

[4] Steen M, Downe S, Bamford N, Edozien L (2012). Not-patient and not-visitor: a metasynthesis fathers’ encounters with pregnancy, birth and maternity care. Midwifery.

[5] Ives J (2014). Men, maternity and moral residue: negotiating the moral demands of the transition to first time fatherhood. Sociol Health Illn.

[6] Rominov H, Giallo R, Pilkington PD, Whelan TA (2018). Getting help for yourself is a way of helping your baby: fathers’ experiences of support for mental health and parenting in the perinatal period. Psychol men Masculinity.

[7] Poh HL, Koh SSL, He HG (2014). An integrative review of fathers’ experiences during pregnancy and childbirth. Int Nurs Rev.

[8] Eggermont K, Beeckman D, Van Hecke A, Delbaere I, Verhaeghe S (2017). Needs of fathers during labour and childbirth: a cross-sectional study. Women Birth: J Australian Coll Midwives.

[9] Locock L, Alexander J (2006). Just a bystander’? Men’s place in the process of fetal screening and diagnosis. Soc Sci Med.

[10] Hildingsson I, Cederlöf L, Widén S (2011). Fathers’ birth experience in relation to midwifery care. Women Birth: J Australian Coll Midwives.

[11] Etheridge J, Slade P (2017). Nothing’s actually happened to me.: the experiences of fathers who found childbirth traumatic. BMC Pregnancy Childbirth.

[12] Wynter K, Di Manno L, Watkins V, Rasmussen B, Macdonald JA (2021). Midwives’ experiences of father participation in maternity care at a large metropolitan health service in Australia. Midwifery.

[13] Beesley A, Karwatzki E, Sullivan K (2019). Anxiety and depression symptoms in fathers during their partner’s pregnancy: how does this impact paternal fetal attachment?. J Prenatal Perinat Psychol Health.

[14] May C, Fletcher R (2013). Preparing fathers for the transition to parenthood: recommendations for the content of antenatal education. Midwifery.

[15] Poh HL, Koh SSL, Seow HCL, He HG (2014). First-time fathers’ experiences and needs during pregnancy and childbirth: a descriptive qualitative study. Midwifery.

[16] Göbel A, Arck P, Hecher K, Schulte-Markwort M, Diemert A, Mudra S (2020). Manifestation and associated factors of pregnancy-related worries in expectant fathers. Front Psychiatry.

[17] Tolman RM, Walsh TB, Fitzgerald HE, von Klitzing K, Cabrera NJ, de Scarano J, Skjøthaug T (2020). Ghosts in the ultrasound: expectant fathers’ experience of trauma. Handbook of fathers and child development.

[18] Ghaffari SF, Elyasi F, Mousavinasab SN, Shahhosseini Z (2021). A systematic review of clinical trials affecting anxiety, stress and fear of childbirth in expectant fathers. Nurs open.

[19] Critchley A (2022). Giving up the ghost: findings on fathers and social work from a study of pre-birth child protection. Qualitative Social work.

[20] Kothari A, Bruxner G, Callaway L, Dulhunty JM (2022). It’s a lot of pain you’ve got to hide: a qualitative study of the journey of fathers facing traumatic pregnancy and childbirth. BMC Pregnancy Childbirth.

[21] Darwin Z, Galdas P, Hinchliff S, Littlewood E, McMillan D, McGowan L (2017). Fathers’ views and experiences of their own mental health during pregnancy and the first postnatal year: a qualitative interview study of men participating in the UK Born and bred in Yorkshire (BaBY) cohort. BMC Pregnancy Childbirth.

[22] Edwards BN, McLemore MR, Baltzell K, Hodgkin A, Nunez O, Franck LS (2020). What about the men? Perinatal experiences of men of color whose partners were at risk for preterm birth, a qualitative study. BMC Pregnancy Childbirth.

[23] Xue WL, Shorey S, Wang W, He HG (2018). Fathers’ involvement during pregnancy and childbirth: an integrative literature review. Midwifery.

[24] Mprah A, Haith-Cooper M, Duda-Mikulin E, Meddings F (2023). A systematic review and narrative synthesis of fathers’ (including migrant fathers’) experiences of pregnancy and childbirth. BMC Pregnancy Childbirth.

[25] Kothari A, Khuu A, Dulhunty J, Bruxner G, Ballard E, Callaway L (2023). Fathers attending the birth of their baby: views, intentions and needs. Australian New Z J Obstet Gynecol.

[26] Connell RW. Masculinities. St. Leonards, Vic. Allen & Unwin; 1995.

[27] Dolan A, Coe C (2011). Men, masculine identities and childbirth. Sociol Health Illn.

[28] Rowe HJ, Holton S, Fisher JRW (2013). Postpartum emotional support: a qualitative study of women’s and men’s anticipated needs and preferred sources. Aust J Prim Health.

[29] Symon AG, Dugard P, Butchart M, Carr V, Paul J (2011). Care and environment in midwife-led and obstetric-led units: a comparison of mothers’ and birth partners’ perceptions. Midwifery.

[30] Fletcher R, May C, St George J, Stoker L, Oshan M. Engaging fathers: Evidence review. Australian Research Alliance for Children and Youth (ARACY). 2014. https://www.aracy.org.au/publications Accessed 15 Jan 2023.

[31] Jeffery T, Luo KY, Kueh B, Petersen RW, Quinlivan JA (2015). Australian fathers’ study: what influences paternal engagement with antenatal care?. J Perinat Educ.

[32] Baldwin S, Malone M, Sandall J, Bick D (2019). A qualitative exploratory study of UK first-time fathers’ experiences, mental health and wellbeing needs during their transition to fatherhood. BMJ open.

[33] Comrie-Thomson L, Gopal P, Eddy K, Baguiya A, Gerlach N, Sauvé C (2021). How do women, men, and health providers perceive interventions to influence men’s engagement in maternal and newborn health? A qualitative evidence synthesis. Soc Sci Med.

[34] Jahanfar S, Howard LM, Medley N. Interventions for preventing or reducing domestic violence against pregnant women. Cochrane database of systematic reviews. 2014. Art. no.: CD009414. 10.1002/14651858.CD009414.pub3. Accessed 01 May 2023. 10.1002/14651858.CD009414.pub3 PMC7104547 25390767

[35] Australian Government Department of Families, Housing, Community Services and Indigenous Affairs. Father-inclusive practice guide: A tool to support the inclusion of fathers in a holistic approach to service delivery. 2009. https://www.dss.gov.au/our-responsibilities/families-and-children/publicationsarticles/father-inclusive-practice-guide. Accessed Aug 29 2022.

[36] Brown H, Davidson D, Ellins J. NHS West Midlands investing for health real-time patient feedback project. Birmingham Health Services Management Centre, University of Birmingham. 2009. https://www.birmingham.ac.uk/Documents/college-social-sciences/social-policy/HSMC/publications/2011/real-time-patient-feedback.pdf. Accessed 16 May 2022.

[37] Bradshaw C, Atkinson S, Doody O. Employing a qualitative description approach in health care research. Global Qualitative Nurs Res. 2017;4. 10.1177/2333393617742282. 10.1177/2333393617742282 PMC5703087 29204457

[38] DeJonckheere M, Vaughn LM (2019). Semistructured interviewing in primary care research: a balance of relationship and rigour. Family Med Community Health.

[39] Leech BL (2002). Asking questions: techniques for semistructured interviews. PS Political Sci Politics.

[40] Saldaña J (2009). The coding manual for qualitative researchers.

[41] Skjott Linneberg M, Korsgaard S (2019). Coding qualitative data: a synthesis guiding the novice. Qualitative Res J.

[42] Soklaridis S, Ravitz P, Adler Nevo G, Lieff S (2016). Relationship-centred care in health: a 20-year scoping review. Patient Experience J.

[43] Robert G, Cornwell J (2013). Rethinking policy approaches to measuring and improving patient experience. J Health Serv Res Policy.

[44] Morse JM, Mitcham C (2002). Exploring qualitatively-derived concepts: inductive—deductive pitfalls. Int J Qualitative Methods.

