# Supplementary material for: Promoting men-inclusive maternity services: exploring the expectations, experiences and needs of men as fathers
Source: BMC Pregnancy Childbirth. 2024 Jul 12;24:477. doi: 10.1186/s12884-024-06644-3 (PMC11245863; doi:10.1186/s12884-024-06644-3)
Supplement: Supplementary file 1 — Supplementary Material 1 [file 12884_2024_6644_MOESM1_ESM.docx]

**Additional file 1: Interview Guides**

**Interview Guide – Pre-birth**

**Recruitment site** (*circle one)*

Maternity Outpatients Department

SHADES clinic

Antenatal classes

Screening appointment

Other __________________________________

**Topics and prompts**

1. What brings you here today?

***Prompts:***

- *Is this your first appointment? When is your baby due?*
- *Did you experience any difficulty getting here today (e.g. getting time off work, childcare, other commitments)?*
- *Do you plan to attend future appointments/classes with your partner? Why or why not?*

1. Tell me a little about yourself (background and demographics).

***Prompts:***

- *Do you live locally or outside Brisbane?*
- *Is this your first baby? Other children?*
- *What is your age range (18-22, 23-27, 28-32, 33-37,38-42, etc)?*
- *Are you currently working? What do you do?*
- *What about your education? Highest level?*

1. Tell me about your experience to date of attending RBWH maternity services?

***Prompts:***

- *As a man, how do you feel about being in maternity services?*
- *Did you feel welcomed? Why or why not?*
- *Did you feel included (e.g in discussions)? What happened? How did that make you feel?*
- *As a man, what are your impressions of the environment (e.g. posters, pictures, décor, written information, magazines)?*
- *Do you feel comfortable returning to the hospital? Why or why not?*

1. Thinking about your own needs as a man, what expectations do you have of maternity services at RBWH?

***Prompts:***

- *Appointments?*
- *Antenatal classes?*
- *Written resources?*
- *Birth/labour?*
- *Discussions with medical staff?*

1. Any other comments?

**Interview Guide – Post-birth**

**Recruitment site** (*circle one)*

Maternity ward

Nursery

Other __________________________________

**Topics and prompts**

1. Tell me about your experience of your baby’s birth.

***Prompts:***

- *Was this your first birth?*
- *Were you present for the birth? How was this for you as the father?*
- *Did you feel included? Why or why not?*

1. Tell me about your expectations of the birth prior to your baby’s birth?

***Prompts:***

- *Did you have any concrete expectations of the birth?*
- *Were your expectations met? Why or why not?*

1. Reflecting on your contact with maternity services prior to the birth, how would you describe your experience?

***Prompts:***

- *Appointments?*
- *Antenatal classes?*
- *Written resources?*
- *Birth/labour?*
- *Discussions with medical staff?*

1. Thinking back on the time that you have been attending RBWH maternity services, is there anything that would have helped you feel more included as a man/father?

***Prompts:***

- *Physical environment?*
- *Written information?*
- *Interactions with staff?*
- *Appointments (e.g. booking in appointments)?*
- *Antenatal classes?*
- *Parent Education?*

1. Any other comments?
