# Supplementary material for: Promoting men-inclusive maternity services: exploring the expectations, experiences and needs of men as fathers
Source: BMC Pregnancy Childbirth. 2024 Jul 12;24:477. doi: 10.1186/s12884-024-06644-3 (PMC11245863; doi:10.1186/s12884-024-06644-3)
Supplement: Supplementary file 2 — Supplementary Material 2 [file 12884_2024_6644_MOESM2_ESM.docx]

**Additional file 2: Development of codes and overarching themes**

**Colour Coding and exemplar quotes**

**Turquoise – Views his role purely as a support person**

*I was there to support Mum.*

*There’s not much I can really do, just support [wife’s name]. It’s her body kind of thing.*

*I’m a very important part of keeping my wife happy and content.*

**Green – Expresses feelings of helplessness as a male**

*Felt helpless as a male.*

*Just nothing you can do, it’s your partner and your baby, it’s their struggle at the time, and there’s nothing you can do to make anything better.*

*There’s not much I can really do, I just have to do what I can.*

**Yellow – Information needs met/felt free to ask questions**

*If I asked questions, they’d always have the answer.*

*You know, she [doctor] was really clear with me, even to me, with what was going on.*

*So they’re just not asking my wife, they’re asking me as well, which is really good.*

**Pink – If my partner and/or child’s needs are met, my needs are met**

*I don't think the staff could have done anything, no. I think their main focus should be the wife, my wife, and that was handled, and I don't think I needed any more.*

*As long as my wife’s happy, I’m pretty happy.*

*Okay, so dad’s needs. If there was anything, I don’t think I needed more of, but I think what was great, that if I was going to ask something for my wife that it was gotten for me and all that. I did have a good experience with that, and that definitely settles the dads down.*

**Red – Observed that staff were responsive and/or approachable**

*You have friendly chats with them all the time, they don’t seem to ignore you or anything. They involve you as much as they can really. I think my experiences have been pretty awesome.*

*The team is really nice here.*

*I felt staff have been over and above my expectations…they have met my needs and my wife’s.*

**Dark grey – Fathers have a responsibility to be present and/or involved.**

*Staff did as much as they could to include me, but it is more about the dad’s responsibility to be present.*

*But the fact that we're going through a pandemic and being a father during this time, all that's happening. I feel there's still great full access for me to be participating in. If there's anything limited in this whole time, it would fall back on me as a father. But I feel personally, the hospital and the staff are giving this window, this timeframe, and it's really up to you as a father to utilise and take it.*

**Light grey – Refers to the difficulties of juggling responsibilities (e.g. other children at home, work)**

*I think I stayed for longer times with [name of first child] too. This time around it’s just I’ve got the kid at home. So, I’ve got to go deal with that as well. So, I’m not here for as long as I’d like to be.*

*I didn’t attend any appointments myself, just she came up. And I was usually at work.*

*So I’ve been here a couple of times for the midwife appointments. I probably came to about half of them, just depending on work and that.*

**Teal – Being directly addressed by staff promoted feelings of inclusion**

*She asked me that about four or five times, and that’s even seeing her just walking out the door and she’d stop, ‘how’s mum, how’s baby, how are you?’ You know, that definitely makes you feel included as a Dad.*

*Mum was the main one, but they always asked me how I was going.*

*it’s not all, “Mum, do this. Mum, do that.” “Dad, you get in and do it” sort of thing.*

**Dark yellow – Inclusion equates with involvement with practical tasks (e.g. cutting the cord, weighing the baby, making or attending appointments) and/or being physically present.**

*I got to cut the umbilical cord, so I was included in that part of it.*

*From my experience, I like to get in there and help.*

*Yes. It’s been great. I can come visit and stay for as long as I want to outside of visiting hours.*

**Overarching themes and subthemes**

*
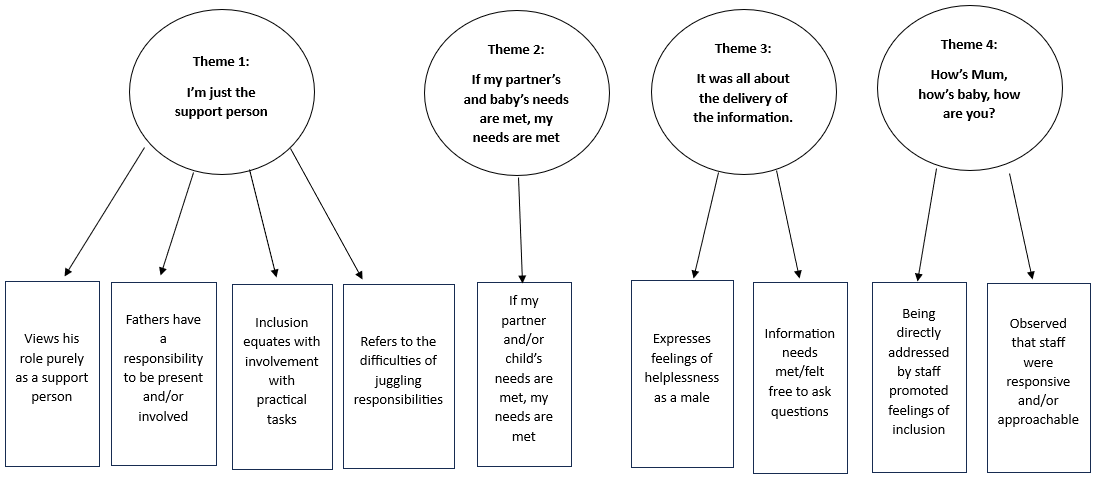
*
